# Supplementary material for: Parental emotional neglect and academic procrastination: the mediating role of future self-continuity and ego depletion
Source: PeerJ. 2023 Oct 23;11:e16274. doi: 10.7717/peerj.16274 (PMC10601898; doi:10.7717/peerj.16274)
Supplement: Supplemental Information 1 [file peerj-11-16274-s001.docx]

Gender: 0=female, 1=male

Homeplace: city=1, rural=2

Family economic situation:

1= very poor, 2= a little poor, 3= average, 4= a little rich, 5= very rich.
