# Supplementary material for: Parental emotional neglect and academic procrastination: the mediating role of future self-continuity and ego depletion
Source: PeerJ. 2023 Oct 23;11:e16274. doi: 10.7717/peerj.16274 (PMC10601898; doi:10.7717/peerj.16274)
Supplement: Supplemental Information 3 [file peerj-11-16274-s003.docx]

**Parental neglect scale:** This scale has been published with presenting all items in Chinese (Deng et al., 2007; Pan, 2006), we have cited these references.

**Future self-continuity**: This scale has only one item, and have been presented in previous research. We have cited this reference (Liu, et al., 2018).

**Ego depletion:** This scale has been published with presenting all items (Johnson & Joanna, 2015), we have cited these references (Lanaj, Johnson, & Barnes, 2014; Johnson & Joanna, 2015).

**Academic procrastination scale**: This scale has been published with presenting all items in Chinese, we have cited this reference (Zheng, 2019).

**All items of these scales were present publicly, we think we could use these instruments.**
